# COVID-19 vaccine hesitancy in Latin America and Africa: a scoping review

**DOI:** 10.1590/0102-311XEN041423

**Published:** 2023-08-07

**Authors:** Bruna Aparecida Gonçalves, Camila Carvalho de Souza Amorim Matos, Jonathan Vicente dos Santos Ferreira, Renata Fortes Itagyba, Vinicius Rocha Moço, Marcia Thereza Couto

**Affiliations:** 1 Faculdade de Medicina, Universidade de São Paulo, São Paulo, Brasil.; 2 Universidade Federal de Santa Catarina, Florianópolis, Brasil.

**Keywords:** Vaccination Hesitancy, COVID-19 Vaccines, Global Health, Hesitação Vacinal, Vacinas Contra COVID-19, Saúde Global, Vacilación a la Vacunación, Vacunas Contra la COVID-19, Salud Global

## Abstract

Vaccination has played an important role in the containment of COVID-19 pandemic advances. However, SARS-CoV-2 vaccine hesitancy has caused a global concern. This scoping review aims to map the scientific literature on COVID-19 vaccine hesitancy in Latin America and Africa from a Global Health perspective, observing the particularities of the Global South and using parameters validated by the World Health Organization (WHO). The review reporting observes the recommendations of the PRISMA for Scoping Reviews (PRISMA-ScR) model. Search was conducted in PubMed, Scopus, Web of Science, and Virtual Health Library (VHL) databases, selecting studies published from January 1, 2020 to January 22, 2022. Selected studies indicate that COVID-19 vaccine hesitancy involves factors such as political scenario, spread of misinformation, regional differences in each territory regarding Internet access, lack of access to information, history of vaccination resistance, lack of information about the disease and the vaccine, concern about adverse events, and vaccine efficacy and safety. Regarding the use of conceptual and methodology references from the WHO for vaccine hesitancy, few studies (6/94) use research instruments based on these references. Then, the replication in Global South of conceptual and methodological parameters developed by experts from the Global North contexts has been criticized from the perspective of Global Health because of it may not consider political and sociocultural particularities, the different nuances of vaccine hesitancy, and issues of access to vaccines.

## Resumo

A vacinação tem papel relevante para conter os avanços da pandemia de COVID-19. No entanto, a hesitação vacinal com os imunizantes que agem contra o SARS-CoV-2 tem causado preocupação em âmbito global. Esta revisão de escopo tem como objetivo mapear a literatura científica sobre a hesitação vacinal contra a COVID-19 na América Latina e África sob uma perspectiva da Saúde Global, observando as particularidades do Sul Global e o uso de parâmetros validados pela Organização Mundial da Saúde (OMS). O relato da revisão segue as recomendações do protocolo PRISMA para Revisões de Escopo (PRISMA-ScR). O levantamento foi realizado nas bases de dados PubMed, Scopus, Web of Science e Biblioteca Virtual em Saúde (BVS), selecionando estudos publicados entre 1º de janeiro de 2020 e 22 de janeiro de 2022, os quais indicam que a hesitação vacinal contra a COVID-19 envolve fatores como o cenário político, a disseminação de desinformação, diferenças regionais referentes ao acesso à Internet, falta de acesso à informação, o histórico de resistência à vacinação, falta de informações sobre a doença e a vacina, preocupação com eventos adversos, eficácia e segurança dos imunizantes. Quanto ao uso dos referenciais conceituais e metodológicos da OMS sobre hesitação vacinal, poucos estudos (apenas 6 de 94) utilizam instrumentos de pesquisa baseado neles. Desta forma, a replicação de parâmetros conceituais e metodológicos elaborados por *expertises* do Norte Global em contextos do Sul Global tem sido criticada pela perspectiva da Saúde Global, em decorrência da possibilidade de não considerar as especificidades políticas e socioculturais, as diferentes nuances de hesitação vacinal e questões de acesso às vacinas.

## Resumen

La vacunación tiene un papel relevante para frenar los avances de la pandemia de COVID-19. Sin embargo, la indecisión a las vacunas contra el SARS-CoV-2 ha causado preocupación a nivel global. Esta revisión de alcance tiene como objetivo mapear la literatura científica sobre la indecisión a las vacunas contra COVID-19 en América Latina y África desde una perspectiva de la Salud Global, observando las particularidades del Sur Global y el uso de parámetros validados por la Organización Mundial de la Salud (OMS). El informe de la revisión sigue las recomendaciones del protocolo PRISMA para Revisiones de Alcance (PRISMA-ScR). La encuesta se realizó en las bases de datos PubMed, Scopus, Web of Science e Biblioteca Virtual en Salud (BVS), seleccionando los estudios publicados entre 1º de enero de 2020 y 22 de enero de 2022. Los estudios seleccionados indican que la indecisión a las vacunas de COVID-19 involucra factores como el escenario político, la diseminación de desinformación, las diferencias regionales de cada territorio referente al acceso a Internet, la falta de acceso a la información, el historial de resistencia a la vacunación, la falta de informaciones sobre la enfermedad y la vacuna, la preocupación por los eventos adversos, la eficacia y la seguridad de los inmunizantes. En cuanto al uso de los referenciales conceptuales y metodológicos de la Organización Mundial de la Salud (OMS) sobre la indecisión a las vacunas, pocos estudios (6/94) utilizan instrumentos de investigación basados en esos referenciales. Así, la replicación de parámetros conceptuales y metodológicos elaborados por expertos del Norte Global en contextos del Sur Global ha sido criticada por la perspectiva de la Salud Global, por la posibilidad de no considerar las especificidades políticas y socioculturales, los diferentes matices de la indecisión a las vacunas y cuestiones de acceso a las vacunas.

## Introduction

The COVID-19 pandemic has exacerbated a complex Global Health scenario with the interaction of the SARS-CoV-2 and noncommunicable diseases, problems of health service access and functioning, socioeconomic inequality, and non-enforcement of social rights, making it a phenomenon of syndemic ^1^.

In addition to health measures such as physical distancing and hygiene, vaccination against COVID-19 significantly contributed to prevent the spread of the epidemic ^2^. A successful vaccine campaign is directly related to the broad acceptance by the population and its effectiveness depends on sustained adoption to maintain the effect of immunity and stop the circulation of the infectious agent ^3^. Despite knowledge legitimized by science about the effectiveness and success of mass immunization, social reactions against vaccines are seen in the history of immunization, creating challenges to Public Health ^4^.

Considering the importance of understanding and implementing actions to address this phenomenon, the working group on vaccine hesitancy of the Strategic Advisory Group of Experts on Immunization (SAGE), World Health Organization (WHO), defined vaccine hesitancy as the “*delay in acceptance or refusal of vaccines despite availability of vaccination services*” ^5^ (p. 7). This definition excludes access issues ^5^ ^,^ ^6^, because “*in low uptake situations where lack of available services is the major factor, hesitancy can be present but is not the principle reason for unvaccinated and undervaccinated members of the community*” ^5^ (p.7).

The WHO EURO Vaccine Communications Working Group proposed the 3C model (confidence, complacency, and convenience), based on the European experience with vaccine hesitancy, which was later reformulated into the 5C scale to include “risk calculation” and “collective responsibility” besides the three determinants of vaccine hesitancy present in the 3C model ^7^. The Matrix of Vaccine Hesitancy Determinants was created to guide the development of vaccine hesitancy indicators, research questions, diagnosis, and intervention ^5^ ^,^ ^6^ ^,^ ^8^. The determinants are grouped into contextual, individual, and group influences/vaccine-specific issues ^5^ ^,^ ^6^ ^,^ ^8^. It is not known whether this matrix was developed from the experiences and aspects of the Global North and South ^9^, but it has been recommended for studies at a global level and studies conducted in the Global South.

More recently, the Working Group on Behavioral and Social Drivers of Vaccination (BeSD), also linked with WHO, has developed another tool to understand the drivers and obstacles to vaccine uptake. The extensive document titled *Behavioural and Social Drivers of Vaccination: Tools and Practical Guidance for Achieving High Uptake* ^10^ contains surveys to investigate determinants of vaccine hesitancy, both in children and specifically regarding COVID-19 vaccines.

In the case of vaccination against COVID-19, studies conducted in African and Latin American countries showed that hesitancy was linked with religious beliefs, association between vaccination and surveillance of government authorities, lack of information about adverse events, vaccine safety and efficacy, and dissemination of fake news ^11^ ^,^ ^12^ ^,^ ^13^ ^,^ ^14^ ^,^ ^15^.

Previous scoping reviews sought to map COVID-19 vaccine hesitancy worldwide ^16^ and in high-income countries ^17^. The results showed aspects related to hesitancy ^16^ ^,^ ^17^: concerns about vaccine safety and efficacy, adverse events, perception of low risk in relation to COVID-19 infection, religious beliefs, cost of vaccine, rapid development of vaccines, lack of trust in government and health authorities, dissemination of fake information, unavailability of clear information about vaccines, racism and discrimination, preference for alternative treatments to the biomedical paradigm.

However, the method strategy of both studies only included publications in English ^16^ ^,^ ^17^. Also, both reviews did not analyze the use of conceptual and methodological tools produced in the Global North applied to Global South countries. Then, our review conducted a reflective analysis on the realities of local contexts of the Global South, with a focus on how the frameworks proposed by WHO SAGE have been used in the Global Health in order to understand the phenomenon of COVID-19 vaccine hesitancy and the impact on health policies ^18^ ^,^ ^19^ ^,^ ^20^.

In this sense, this scoping review intends to promote original contributions to the particularities of social, cultural, and local aspects of COVID-19 vaccine hesitancy in Latin American and African countries from a critical perspective of Global Health ^21^, which consider the relations of power, authority, inclusion and exclusion observed in the scientific field, governments, and health institutions in the Global North and Global South. This perspective highlights inequalities among actors who design and actors who receive global health interventions, in order to understand the reproduction of the dichotomy between “the West and the rest” ^18^.

Vaccine hesitancy in the Global South must be understood according to the complexity of cultural, social, ethnic, and regional differences ^9^, including vaccines against COVID-19. Then, this study aims to identify, map, and systematize scientific evidence of COVID-19 vaccine hesitancy in Latin American and African countries.

## Method

This scoping review seeks to understand broader issues in order to synthesize evidence and map the literature about a field of knowledge that has not yet been fully reviewed or has a complex and heterogeneous nature ^22^ ^,^ ^23^. This study is based on the following question: How has the scientific literature addressed COVID-19 vaccine hesitancy in Latin American and African countries?

This scoping review reporting was structured according to the PRISMA for Scoping Reviews (PRISMA-ScR) checklist items ^22^ ^,^ ^23^: title, structured summary, rationale, objectives, methods (review protocol, eligibility criteria, information sources, search, selection of sources of evidence, organization and synthesis of results), results (selection of evidence, characteristics, appraisal, presentation, and synthesis of results), discussion according to critical global health perspective, study limitations, and final considerations.

Studies in English, Portuguese, and Spanish published from January 1st, 2020 (year when COVID-19 was considered a Public Health Emergency of International Concern by the WHO) to January 22, 2022 were included in this review. A search was conducted in PubMed, Scopus, Web of Science, and Virtual Health Library (VHL) databases. Eligibility criteria included complete empirical, qualitative, quantitative, mixed methods research studies that explicitly and implicitly include COVID-19 vaccine hesitancy in their results, indicating outcomes of acceptance or not, performed with any population in Latin American and African countries, regardless of age group, gender, or other criteria of social differentiation. Publications such as comments, editorials, studies on COVID-19 vaccine development, reviews, studies that did not cover countries in Africa or Latin America, and studies that did not include findings on COVID-19 vaccine hesitancy in their results and discussions were not included.

Searches in the databases were performed in January 2022 using descriptors related to COVID-19, vaccine hesitancy, and countries in Latin America and/or Africa (Supplementary Material: https://cadernos.ensp.fiocruz.br/static//arquivo/suppl-e00041423-en_8375.pdf). Search results were exported to the EndNote (https://endnote.com/) bibliographic reference manager and duplicate studies were excluded. After that, the main author read the titles and abstracts of all studies to exclude those that did not meet the eligibility criteria. In case of any doubt, a second reviewer performed the arbitration by reading the title and abstract, and if doubts persisted, the full study was read.

Three aspects guided the extraction of information in the study reading stage, which were inserted into a Microsoft Excel (https://products.office.com) spreadsheet: (1) General characterization of the studies, including authors, year of publication, journal, country of affiliation, institution of the corresponding author, method aspects (country where the investigation was conducted, study population, objective, and design); (2) Study results regarding acceptance hesitancy, and related reasons; (3) Information of the reference (or not) to the concepts and method references of the WHO SAGE and the context-specific particularities of the Global South reported in the studies. Then, an interpretative analysis of these findings was conducted using the critical perspective in global health regarding vaccine hesitancy ^18^ ^,^ ^21^.

## Results

### General characteristics of the studies

**Figure 1:**
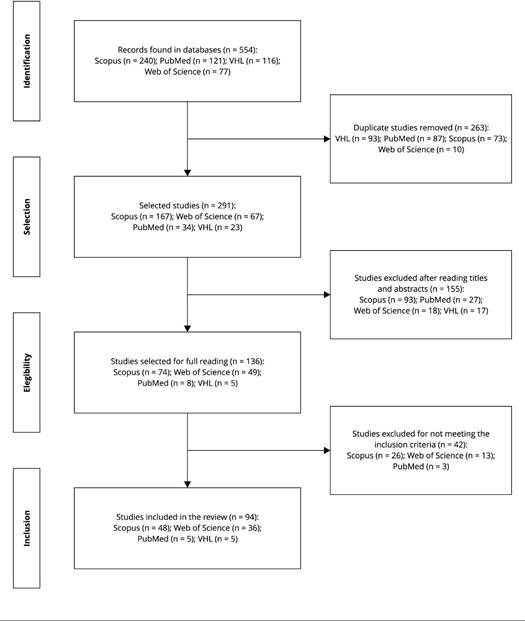
Flowchart identifying the studies included in this scoping review.

After the stages of search and study selection, 94 studies were included in this review. Figure 1 shows a flowchart of these stages.

VHL: Virtual Health Library. Source: prepared by the authors.

Regarding the general characteristics of the studies, considering database search was performed in January 2022, most studies were published in 2021 (89), and conducted in African countries (62) followed by Latin American countries (20). Multicenter studies (12) were the most predominant design. Regarding the countries of affiliation, in African studies, most common countries producing university studies were Ethiopia (14), Nigeria (14), Ghana (7), South Africa (6), and Egypt (6). In Latin America, the Brazilian institutions were more commonly found (6). However, some publications had corresponding authors linked with institutions in the United Arab Emirates (3) and France (2). Multicenter studies had corresponding authors affiliated with institutions from countries in the Global North - the United States (6), Belgium (2), and the United Kingdom (2). Box 1 shows the studies selected for this review.

**Box 1:** Studies selected for this scoping review according to title, authors, year of publication, study place, and institution of corresponding author.

TITLE	AUTHORS	YEAR	STUDY PLACE	INSTITUTION OF CORRESPONDING AUTHOR
*The Coronavirus Disease 2019 (COVID-19) Vaccination Psychological Antecedent Assessment Using the Arabic 5c Validated Tool: An Online Survey in 13 Arab Countries*	Abdou et al. ^66^	2021	United Arab Emirates, Saudi Arabia, Kuwait, Syria, Lybia, Lebanon, Sudan, Jordan, Iraq, Morocco and Egypt	Epidemiology Department, High Institute of Public Health, Alexandria University (Alexandria, Egypt)
*Examining Vaccine Hesitancy in Sub-Saharan Africa: A Survey of the Knowledge and Attitudes among Adults to Receive COVID-19 Vaccines in Ghana*	Acheampong et al. ^11^	2021	Ghana	iRIS Research Consortium (Accra, Ghana)
*Guarding Against COVID-19 Vaccine Hesitancy in Ghana: Analytic View of Personal Health Engagement and Vaccine Related Attitude*	Addo et al. ^95^	2021	Ghana	Applied Sciences and Mathematical Education, Akenten Appiah-Menka University of Skills Training and Entrepreneurial Development (Kumasi, Ghana)
*When It is Available, Will We Take It? Social Media Users’ Perception of Hypothetical COVID-19 Vaccine in Nigeria*	Adebisi et al. ^44^	2021	Nigeria	Department of Clinical Pharmacy and Pharmacy Administration, Faculty of Pharmacy, University of Ibadan (Ibadan, Nigeria)
*Perceptions of the COVID-19 Vaccine and Willingness to Receive Vaccination among Health Workers in Nigeria*	Adejumo et al. ^96^	2021	Nigeria	Department of Community Medicine, University of Medical Sciences (Ondo City, Nigeria)
*Acceptance of COVID-19 Vaccine among the Healthcare Workers in the Eastern Cape, South Africa: A Cross Sectional Study*	Adeniyi et al. ^97^	2021	South Africa	Department of Family Medicine and Rural Health, Walter Sisulu University, Cecilia Makiwane Hospital, East London Hospital Complex (East London, South Africa)
*COVID-19 Vaccine Hesitancy and Willingness to Pay: Emergent Factors from a Cross-Sectional Study in Nigeria*	Adigwe ^26^	2021	Nigeria	National Institute for Pharmaceutical Research and Development (Abuja, Nigeria)
*Determinants of COVID-19 Vaccine Hesitancy among Health Care Workers in Amhara Region Referral Hospitals, Northwest Ethiopia: A Cross-Sectional Study*	Aemro et al. ^98^	2021	Ethiopia	Department of Medical nursing, School of Nursing, College of Medicine and Health Science, University of Gondar (Gondar, Ethiopia)
*Drivers of COVID-19 Vaccine Uptake amongst Healthcare Workers (HCWs) in Nigeria*	Agha et al. ^99^	2021	Nigeria	Global Delivery Program, Bill & Melinda Gates Foundation (Seattle, United States)
*Acceptability of COVID-19 Vaccination among Health Care Workers in Ghana*	Agyekum et al. ^52^	2021	Ghana	Department of Sociology and Social Work, Kwame Nkrumah University of Science and Technology (Kumasi, Ghana)
*COVID-19 Vaccine Acceptability and Adherence to Preventive Measures in Somalia: Results of an Online Survey*	Ahmed et al. ^37^	2021	Somalia	Global Health Institute, University of Antwerp (Antwerp, Belgium)/Brain Research Africa Initiative (BRAIN) (Yaounde, Cameroon)
*COVID-19 Vaccine Hesitancy among the Adult Population in Ghana: Evidence from a Pre-Vaccination Rollout Survey*	Alhassan et al. ^32^	2021	Ghana	Centre for Health Policy and Implementation Research, Institute of Health Research, University of Health and Allied Sciences (Ho, Ghana)
*COVID-19 Vaccine Uptake among Health Care Workers in Ghana: A Case for Targeted Vaccine Deployment Campaigns in the Global South*	Alhassan et al. ^55^	2021	Ghana	Institute of Health Research, University of Health and Allied Sciences (Ho, Ghana)
*Attitude and Associated Factors of COVID-19 Vaccine Acceptance among Health Professionals in Debre Tabor Comprehensive Specialized Hospital, North Central Ethiopia; 2021: Cross-Sectional Study*	Alle & Oumer ^100^	2021	Ethiopia	Department of Anesthesia, College of Health Sciences, School of Medicine, Debre Tabor University (Debre Tabor, Ethiopia)
*Perception of COVID‐19 Vaccination amongst Physicians in Colombia*	Alvarado-Socarras et al. ^101^	2021	Colombia	Colombian Cardiovascular Foundation (Floridablanca, Colombia)
*COVID-19 Vaccine Hesitancy among Healthcare Workers and Its Socio-Demographic Determinants in Abia State, Southeastern Nigeria: A Cross-Sectional Study*	Amuzie et al. ^102^	2021	Nigeria	Department of Community Medicine, Federal Medical Centre (Umuahia, Nigeria)
*Vaccine Hesitancy and Religiosity in a Sample of University Students in Venezuela*	Andrade ^103^	2021	Venezuela	College of Medicine, Ajman University (Ajman, United Arab Emirates)
*COVID-19 Vaccine Hesitancy, Conspiracist Beliefs, Paranoid Ideation and Perceived Ethnic Discrimination in a Sample of University Students in Venezuela*	Andrade ^42^	2021	Venezuela	Ajman University (Ajman, United Arab Emirates)
*Predictive Demographic Factors of COVID-19 Vaccine Hesitancy in Venezuela: A Cross-Sectional Study*	Andrade ^43^	2021	Venezuela	Ajman University (Ajman, United Arab Emirates)
*Health Care Workers Intention to Accept COVID-19 Vaccine and Associated Factors in Southwestern Ethiopia, 2021*	Angelo et al. ^64^	2021	Ethiopia	Department of Nursing, Mizan-Tepi University (Mizan-Aman, Ethiopia)
*Will Africans Take COVID-19 Vaccination?*	Anjorin et al. ^24^	2021	Multinational/Africa	Department of Microbiology (Virology Research), Lagos State University (Lagos, Nigeria)
*Communicating COVID-19 Vaccine Safety: Knowledge and Attitude among Residents of South East, Nigeria*	Anorue et al. ^104^	2021	Nigeria	Department of Mass Communication, University of Nigeria (Nsukka, Nigeria)
*To Get Vaccinated or Not? Social Psychological Factors Associated with Vaccination Intent for COVID-19*	Baeza-Rivera et al. ^105^	2021	Chile	Psychology Department, Faculty of Health Sciences, Temuco Catholic University (Temuco, Chile)
*COVID-19 Vaccine Hesitancy among Parents of Children and Adolescents Living in Brazil*	Bagateli et al. ^61^	2021	Brazil	Department of Clinical Science and Community Health, University of Milan (Milan, Italy)
*Acceptance of COVID-19 Vaccine and Determinant Factors among Patients with Chronic Disease Visiting Dessie Comprehensive Specialized Hospital, Northeastern Ethiopia*	Berihun et al. ^106^	2021	Ethiopia	Department of Environmental Health, College of Medicine and Health Sciences, Wollo University (Dessie, Ethiopia)
*COVID-19 Vaccine Acceptance among High-Risk Populations in Uganda*	Bongomin et al. ^53^	2021	Uganda	Department of Medical Microbiology and Immunology, Faculty of Medicine, Gulu University (Gulu, Uganda)/Department of Medicine, School of Medicine, Makerere University College of Health Sciences (Kampala, Uganda)
*Factors Affecting COVID-19 Vaccine Acceptance: An International Survey Among Low- and Middle-Income Countries*	Bono et al. ^36^	2021	Brazil, Malaysia, Thailand, Bangladesh, Democratic Republic of Congo, Benin, Uganda, Malawi and Mali	Centre for Community Health Studies (ReaCH), Faculty of Health Sciences, Universiti Kebangsaan Malaysia (Kuala Lumpur, Malaysia)
*COVID-19 Vaccine Hesitancy Concerns: Findings from a Ghana Clinical Radiography Workforce Survey*	Botwe et al. ^59^	2021	Ghana	Department of Radiography, School of Biomedical and Allied Health Sciences, College of Health Sciences, University of Ghana (Accra, Ghana)
*COVID-19 Vaccine Hesitancy in Zambia: A Glimpse at the Possible Challenges Ahead for COVID-19 Vaccination Rollout in Sub-Saharan Africa*	Carcelen et al. ^50^	2021	Zambia	Department of International Health, International Vaccine Access Center, Johns Hopkins Bloomberg School of Public Health (Baltimore, United States)
*Hesitation and Refusal Factors in Individuals’ Decision-Making Processes Regarding a Coronavirus Disease 2019 Vaccination*	Cerda & García ^39^	2021	Chile	Faculty of Economics and Business, University of Talca (Talca, Chile)
*Hesitation Regarding the COVID-19 Vaccine among Medical Students in Brazil*	Chaves et al. ^47^	2021	Brazil	Cariri Federal University (Barbalha, Brazil)
*Maternal Level of Awareness and Predictors of Willingness to Vaccinate Children against COVID 19; A Multi-Center Study*	Chinawa et al. ^107^	2021	Nigeria	Department of Pediatrics, College of Medicine, University of Nigeria (Enugu, Nigeria)
*Unmasking COVID-19 Vaccine “Infodemic” in the Social Media*	Demuyakor et al. ^108^	2021	Ghana	Institute of Communication Studies, Communication University of China (Beijing, China)
*Assessment of Vaccine Hesitancy to a COVID-19 Vaccine in Cameroonian Adults and its Global Implication*	Dinga et al. ^65^	2021	Cameroon	Biotechnology Unit, Faculty of Science, University of Buea (Buea, Cameroon)
*Covid-19 Vaccine Acceptance in the Democratic Republic of Congo: A Cross-Sectional Survey*	Ditekemena et al. ^51^	2021	Democratic Republic of Congo	Kinshasa School of Public Health, University of Kinshasa (Kinshasa, Democratic Republic of Congo)
*Levers and Barriers to Vaccinate against COVID-19 in the Multicultural Context of French Guiana: A Qualitative Cross-Sectional Survey among Health Care Workers*	Douine et al. ^109^	2021	French Guiana	Antilles and Guyana Clinical Research Center/Cayenne Hospital Center (Cayenne, French Guiana)
*COVID-19 Vaccine Acceptability and its Determinants in Mozambique: An Online Survey*	Dula et al. ^35^	2021	Mozambique	Global Health Institute, University of Antwerp (Antwerp, Belgium)
*Predictors of COVID-19 Vaccine Hesitancy among Egyptian Healthcare Workers: A Cross-Sectional Study*	El-Sokkary et al. ^110^	2021	Egypt	Medical Microbiology and Immunology Department, Faculty of Medicine, Zagazig University (Ash Sharqiyah, Egypt)
*Knowledge, Attitude, and Acceptance of Healthcare Workers and the Public Regarding the COVID-19 Vaccine: A Cross-Sectional Study*	Elhadi et al. ^34^	2021	Lybia	Faculty of Medicine, University of Tripoli (Tripoli, Lybia)
*Factors Influencing Decision Making Regarding the Acceptance of the COVID-19 Vaccination in Egypt: A Cross-Sectional Study in an Urban, Well-Educated Sample*	Elsayed et al. ^29^	2022	Egypt	Department of Psychiatry and Psychotherapy III, University of Ulm (Ulm, Germany)
*COVID-19 Vaccination Perception and Attitude among Healthcare Workers in Egypt*	Fares et al. ^30^	2021	Egypt	Cairo University (Cairo, Egypt)
*Prevalence and Factors Associated with COVID-19 Vaccine Hesitancy in Health Professionals in Togo, 2021*	Gbeasor-Komlanvi et al. ^111^	2021	Togo	Department of Public Health, University of Lomé (Lomé, Togo)
*When Politics Collides with Public Health: COVID-19 Vaccine Country of Origin and Vaccination Acceptance in Brazil*	Gramacho & Turgeon ^48^	2021	Brazil	Faculty of Communication, University of Brasilia (Brasilia, Brazil)
*Vaccine Hesitancy among Communities in Ten Countries in Asia, Africa, and South America during the COVID-19 Pandemic*	Harapan et al. ^67^	2021	Bangladesh, India, Iran, Pakistan, Egypt, Nigeria, Sudan, Tunisia, Brazil and Chile	Medical Research Unit, School of Medicine, Universitas Syiah Kuala (Banda Achém, Indonesia)
*‘Why Should I Take the COVID-19 Vaccine after Recovering from the Disease?’ A Mixed-Methods Study of Correlates of COVID-19 Vaccine Acceptability among Health Workers in Northern Nigeria*	Iliyasu et al. ^54^	2021	Nigeria	Department of Community Medicine, Bayero University (Kano, Nigeria)
*Predictors of COVID-19 Vaccine Acceptability among Patients Living with HIV in Northern Nigeria: A Mixed Methods Study*	Iliyasu et al. ^112^	2021	Nigeria	Department of Community Medicine, Bayero University (Kano, Nigeria)
*“They Have Produced a Vaccine, but We Doubt if COVID-19 Exists”: Correlates of COVID-19 Vaccine Acceptability among Adults in Kano, Nigeria*	Iliyasu et al. ^113^	2021	Nigeria	Department of Community Medicine, Bayero University (Kano, Nigeria)
*COVID-19 Vaccine Acceptance in Azuay Province, Ecuador: A Cross-Sectional Online Survey*	Jaramillo-Monge et al. ^14^	2021	Ecuador	Family Medicine and Population Health, University of Antwerp (Antwerp, Belgium)
*Acceptance of COVID-19 Vaccines in Sub-Saharan Africa: Evidence from Six National Phone Surveys*	Kanyanda et al. ^25^	2021	Burkina Faso, Ethiopia, Malawi, Mali, Nigeria and Uganda	Development Data Group, World Bank Group (Washington DC, United States)
*Acceptance of the Coronavirus Disease-2019 Vaccine among Medical Students in Uganda*	Kanyike et al. ^60^	2021	Uganda	Faculty of Health Sciences, Busitema University (Mbale, Uganda)
*Trust about Corona Vaccine among Health Professionals Working at Dilla University Referral Hospital, 2021*	Kassaw & Shumye ^114^	2021	Ethiopia	Department of Psychiatry, College of Health Science, Dilla University (Dilla, Ethiopia)
*COVID-19 Vaccination Acceptance among Health Science Students in Morocco: A Cross-Sectional Study*	Khalis et al. ^115^	2021	Morocco	International School of Public Health, Mohammed VI University of Health Sciences (Casablanca, Morocco)
*Acceptability of COVID-19 Vaccination among Health Care Workers: A Cross-Sectional Survey in Morocco*	Khalis et al. ^116^	2021	Morocco	International School of Public Health, Mohammed VI University of Health Sciences (Casablanca, Morocco)
*COVID-19 Vaccination Acceptance and Its Associated Factors among Cancer Patients in Tunisia*	Khiari et al. ^117^	2021	Tunisia	Department of Epidemiology and Biostatistics, Salah Azaiz Institute (Tunis, Tunisia)
*COVID19 Vaccine Intentions in South Africa: Health Communication Strategy to Address Vaccine Hesitancy*	Kollamparambil et al. ^28^	2021	South Africa	University of the Witwatersrand (Johannesburg, South Africa)
*A Nationwide Survey of the Potential Acceptance and Determinants of COVID-19 Vaccines in Ghana*	Lamptey et al. ^56^	2021	Ghana	Institute of Life and Earth Sciences, Pan African University, University of Ibadan (Ibadan, Nigeria)
*Hesitant or Not? The Association of Age, Gender, and Education with Potential Acceptance of a COVID-19 Vaccine: A Country-Level Analysis*	Lazarus et al. ^27^	2020	South Africa, Brazil, Canada, China, North Korea, Ecuador, Spain, United States, France, Germany, India, Italy, Mexico, Nigeria, Poland, United Kingdom, Ressia, Singapore and Sweden	Barcelona Institute for Global Health (ISGlobal), University of Barcelona (Barcelona, Spain)
*Which Older Brazilians Will Accept a COVID-19 Vaccine? Cross-Sectional Evidence from the Brazilian Longitudinal Study of Aging (ELSI-Brazil)*	Macinko et al. ^62^	2021	Brazil	Department of Health Policy and Management, University of California Los Angeles Jonathan and Karin Fielding School of Public Health (Los Angeles, United States)
*COVID-19 Vaccine Hesitancy and Emerging Variants: Evidence from Six Countries*	Mangla et al. ^118^	2021	Bangladesh, Colombia, India, Malaysia, Zimbabwe and United States	International Institute for Population Sciences (Mumbai, India)
*Understanding COVID-19 Vaccine Hesitancy and Resistance: Another Challenge in Cancer Patients*	Mejri et al. ^119^	2020	Tunisia	Medical Oncology Department, Abderrahmane Mami Hospital, Faculty of Medicine, University Tunis El Manar (Tunis, Tunisia)
*COVID-19 Vaccination Acceptance and Its Associated Factors in Sodo Town, Wolaita Zone, Southern Ethiopia: Cross-Sectional Study*	Mesele ^31^	2021	Ethiopia	School of Midwifery, College of Health Science and Medicine, Wolaita Sodo University (Wolaita Sodo, Ethiopia)
*COVID-19 Vaccine Hesitancy among Ethiopian Healthcare Workers*	Mohammed et al. ^120^	2021	Ethiopia	School of Pharmacy, College of Health Sciences, Addis Ababa University (Addis Ababa, Ethiopia)
*Low COVID-19 Vaccine Hesitancy in Brazil*	Moore et al. ^121^	2021	Brazil	Fernandes Figueira National Institute of Women, Children and Adolescents' Health, Oswaldo Cruz Foundation (Rio de Janeiro, Brazil)
*Willingness to Receive COVID-19 Vaccine and Its Determinant Factors among Lactating Mothers in Ethiopia: A Cross-Sectional Study*	Mose ^122^	2021	Ethiopia	Department of Midwifery, College of Medicine and Health Science, Wolkite University (Wolkite, Ethiopia)
*COVID-19 Vaccine Acceptance and Its Associated Factors among Pregnant Women Attending Antenatal Care Clinic in Southwest Ethiopia: Institutional-Based Cross-Sectional Study*	Mose & Yeshaneh ^123^	2021	Ethiopia	Department of Midwifery, College of Medicine and Health Science, Wolkite University (Wolkite, Ethiopia)
*Factors Associated with Acceptance of COVID-19 Vaccine among University Health Sciences Students in Northwest Nigeria*	Mustapha et al. ^57^	2021	Nigeria	School of Pharmaceutical Sciences, Univerisiti Sains Malaysia (Penang, Malaysia)/Department of Clinical Pharmacy and Pharmacy Practice, Faculty of Pharmaceutical Sciences, Ahmadu Bello University (Zaria, Nigeria)
*Estimating Vaccine Confidence Levels among Healthcare Staff and Students of a Tertiary Institution in South Africa*	Oduwole et al. ^124^	2021	South Africa	University of Stellenbosch (Stellenbosch, South Africa)
*Community Acceptance and Willingness to Pay for Hypothetical COVID-19 Vaccines in a Developing Country: A Web-Based Nationwide Study in Nigeria*	Okafor et al. ^125^	2021	Nigeria	Nigeria Board of Pharmacists (Abuja, Nigeria)
*Prevalência e Fatores Associados à Hesitação Vacinal contra a COVID-19 no Maranhão, Brasil*	Oliveira et al. ^85^	2021	Brazil	Federal University of Maranhão (São Luís, Brazil)
*Attitudes and Intentions Towards COVID-19 Vaccines and Associated Factors among Egyptian Adults*	Omar & Hani ^126^	2021	Egypt	Faculty of Medicine, University of Benha (Benha, Egypt)
*Assessing the Level and Determinants of COVID-19 Vaccine Confidence in Kenya*	Orangi et al. ^127^	2021	Kenya	Health Economic Research Unit (Nairobi, Kenya)
*Compliance Indicators of COVID-19 Prevention and Vaccines Hesitancy in Kenya: A Random-Effects Endogenous Probit Model*	Oyekale ^46^	2021	Kenya	North-West University (Mafikeng, South Africa)
*Willingness to Take COVID-19 Vaccines in Ethiopia: An Instrumental Variable Probit Approach*	Oyekale ^45^	2021	Ethiopia	North-West University (Mafikeng, South Africa)
*Running Away from the Jab: Factors Associated with COVID-19 Vaccine Hesitancy in Brazil*	Paschoalotto et al. ^49^	2021	Brazil	Nova University Lisbon (Lisbon, Portugal)
*Hesitancy of Arab Healthcare Workers Towards COVID-19 Vaccination: A Large-Scale Multinational Study*	Qunaibi et al. ^128^	2021	Multinational/International	Jerash Private University (Jerash, Jordan)
*Characteristics Associated with COVID-19 Vaccine Hesitancy: A Nationwide Survey of 1000 Patients with Immune-Mediated Inflammatory Diseases*	Rezende et al. ^129^	2021	Brazil	Fluminense Federal University (Niteroi, Brazil)
*Influence of Health Beliefs on COVID-19 Vaccination among Individuals with Cancer and Other Comorbidities in Puerto Rico*	Rodriguez et al. ^15^	2021	Puerto Rico	Department of Epidemiology, Rollins School of Public Health, Emory University (Atlanta, United States)
*Vaccine Hesitancy: Beliefs and Barriers Associated with COVID-19 Vaccination among Egyptian Medical Students*	Saied et al. ^130^	2021	Egypt	Department of Public Health and Community Medicine, Faculty of Medicine, Tanta University (Tanta, Egypt)
*Factors Influencing COVID-19 Vaccination Demand and Intent in Resource-Limited Settings: Based on Health Belief Model*	Seboka et al. ^131^	2021	Ethiopia	Department of Health Informatics, School of Public Health, College of Health Science and Medicine, Dilla University (Dilla, Ethiopia)
*Physicians’ Attitudes and Acceptance Regarding COVID-19 Vaccines: A Cross-Sectional Study in Mid Delta Region of Egypt*	Shehata et al. ^132^	2021	Egypt	Department of Public Health and Community Medicine, Faculty of Medicine, Tanta University (Tanta, Egypt)
*Exploring Reasons for COVID-19 Vaccine Hesitancy among Healthcare Providers in Ethiopia*	Shiferie et al. ^58^	2021	Ethiopia	Department of Epidemiology and Biostatistics, Addis Continental Institute of Public Health (Adis Abeba, Ethiopia)
*COVID-19 Vaccine Acceptance and Hesitancy in Low- and Middle-Income Countries*	Solís Arce et al. ^133^	2021	Burkina Faso, Colombia, India, Mozambique, Nepal, Nigeria, Pakistan, Rwanda, Sierra Leoa, Uganda, Russia and United States	Yale University (New Haven, United States)
*Global Trends and Correlates of COVID-19 Vaccination Hesitancy: Findings from the iCARE Study*	Stojanovic et al. ^40^	2021	Brazil, Canada, Colombia, France, Italy, Turkey, United Kingdom and United States	Montreal Behavioral Medicine Center, Integrated University Health and Social Services Center of the North Island of Montreal (Montreal, Canada)
*Knowledge and Proportion of COVID-19 Vaccination and Associated Factors among Cancer Patients Attending Public Hospitals of Addis Ababa, Ethiopia, 2021: A Multicenter Study*	Tadele Admasu ^134^	2021	Ethiopia	Department of Biomedical Sciences, College of Health Sciences, Debre Tabor University (Debre Tabor, Ethiopia)
*COVID-19 Vaccination in Lower-Middle Income Countries: National Stakeholder Views on Challenges, Barriers, and Potential Solutions*	Tagoe et al. ^135^	2021	Bangladesh and Ghana	Department of Management Science, University of Strathclyde (Glasgow, United Kingdom)
*Coronavirus Disease 2019 Vaccine Acceptance and Perceived Barriers among University Students in Northeast Ethiopia: A Cross-Sectional Study*	Taye et al. ^136^	2021	Ethiopia	Department of Midwifery, College of Medicine and Health Sciences, Debre Berhan University (Debre Berhan, Ethiopia)
*Willingness to Get the COVID-19 Vaccine among Residents of Slum Settlements*	Ticona et al. ^38^	2021	Brazil	Public Health Institute, Fedeal University of Bahia (Salvador, Brazil)
*COVID-19 Vaccine Hesitancy among Staff and Students in a Nigerian Tertiary Educational Institution*	Uzochukwu et al. ^137^	2021	Nigeria	Department of Pharmaceutical & Medicinal Chemistry, Faculty of Pharmaceutical Sciences, Nnamdi Azikiwe University (Awka, Nigeria)
*Attitudes Towards the COVID-19 Vaccine and Willingness to Get Vaccinated among Healthcare Workers in French Guiana: The Influence of Geographical Origin*	Vignier et al. ^138^	2021	French Guiana	Antilles and Guyana Clinical Research Center/Cayenne Hospital Center (Cayenne, French Guiana)
*Sociodemographic Predictors Associated with the Willingness to Get Vaccinated against COVID-19 in Peru: A Cross-Sectional Survey*	Vizcardo et al. ^41^	2022	Peru	Vice-Rectory for Research, Norbert Wiener University (Lima, Peru)
*COVID-19 Vaccine Acceptance and Hesitancy among Healthcare Workers in South Africa*	Wiysonge et al. ^139^	2022	South Africa	Cochrane South Africa, South African Medical Research Council (Cape Town, South Africa)
*Challenges in Ensuring Global Access to COVID-19 Vaccines: Production, Affordability, Allocation, and Deployment*	Wouters et al. ^140^	2021	Argentina, Brazil, Chile, Ecuador, Nigeria, Paraguay and Peru	Department of Health Policy, London School of Economics and Political Science (London, United Kingdom)
*Knowledge into the Practice against COVID-19: A Cross-Sectional Study from Ghana*	Yeboah et al. ^33^	2021	Ghana	Department of Pharmacy Practice, Faculty of Pharmacy and Pharmaceutical Sciences, Kwame Nkrumah University of Science & Technology (Kumasi, Ghana)
*Intention to Receive the Second Round of COVID-19 Vaccine among Healthcare Workers in Eastern Ethiopia*	Zewude & Belachew ^141^	2021	Ethiopia	Department of Sociology, Wolaita Sodo University (Wolaita Sodo, Ethiopia)

Source: prepared by the authors.

Regarding the study method, most were quantitative studies (85), followed by mixed methods studies (7) and, finally, qualitative studies (2). As for the study population, most were general population (45), followed by health professionals (24), university students (9), individuals with comorbidities (8), health students and professionals (2), university employees and students (1), parents and/or caregivers of children and/or adolescents (4), and population over 50 years old (1).

### COVID-19 vaccine acceptance and hesitancy in a comparative perspective

As the results of studies according to acceptance, hesitancy, and related reasons, most studies presented data of COVID-19 vaccine acceptance (88).

A study conducted across the African continent found population acceptance of 63% ^24^. In Nigeria, studies reported the highest acceptance of 88.5% ^25^ and the lowest acceptance of 22.7% ^26^. In South Africa, studies reported acceptance ranging from 81.6% ^27^ to 55% ^28^. In Egypt, the highest acceptance was 32.85% ^29^ and the lowest, 21% ^30^. In Ethiopia, the highest acceptance was 97.9% ^25^ and the lowest, 45.5% ^31^. In Ghana, acceptance ranged from 64.72% ^32^ to 35% ^33^. In Libya, acceptance ranged from 79.6% to 41.2%, depending on the vaccine efficacy ^34^. Mozambique reported 71.4% acceptance ^35^. In Burkina Faso, acceptance was 79.6% ^25^. In the Democratic Republic of the Congo, the highest and lowest acceptance rates were 59.4% and 32.9% ^36^. In Somalia, acceptance was 76.8% ^37^. In Uganda, depending on the vaccine efficacy, acceptance was 88.8% and 65.4% ^36^. Acceptance in Benin was 48.4% and 22.6%, depending on the vaccine efficacy ^36^. Malawi had acceptance of 61.7% and 44.4%, depending on the vaccine efficacy ^36^. In Mali, it ranged from 74.5% to 45.5%, depending on the vaccine efficacy ^36^.

In Latin American countries, the highest and lowest acceptance rates were 94.2% ^36^ and 66% ^38^ in Brazil. In Ecuador, vaccine acceptance ranged from 91% to 27%, depending on the vaccine efficacy ^14^. In Chile, acceptance was 49% ^39^, and in Colombia acceptance ranged from 71.56% to 57.23% ^40^. In Peru, vaccine acceptance was 70.4% ^41^, and 71.25% in Venezuela ^42^ ^,^ ^43^.

Regarding hesitancy and refusal of COVID-19 vaccines in Africa, the highest hesitancy rate was 52.9% ^26^ and the lowest 25.5% ^44^ in Nigeria. In South Africa, hesitancy was 29.16% ^28^. In Egypt, the highest hesitancy rate was 67.15% ^29^ and the lowest, 28% ^30^. In Ethiopia, the highest hesitancy rate was 54.5% ^31^ and the lowest, 6.61% ^45^ ^,^ ^46^. Burkina Faso had 53.7% refusal ^25^. In Ghana, the highest refusal was 35.28% ^32^ and the lowest, 21% ^11^. In Libya, refusal ranged from 58.8% to 20.4%, depending on the vaccine efficacy ^34^. Mozambique had 28.6% refusal rate ^35^.

**Figure 2:**
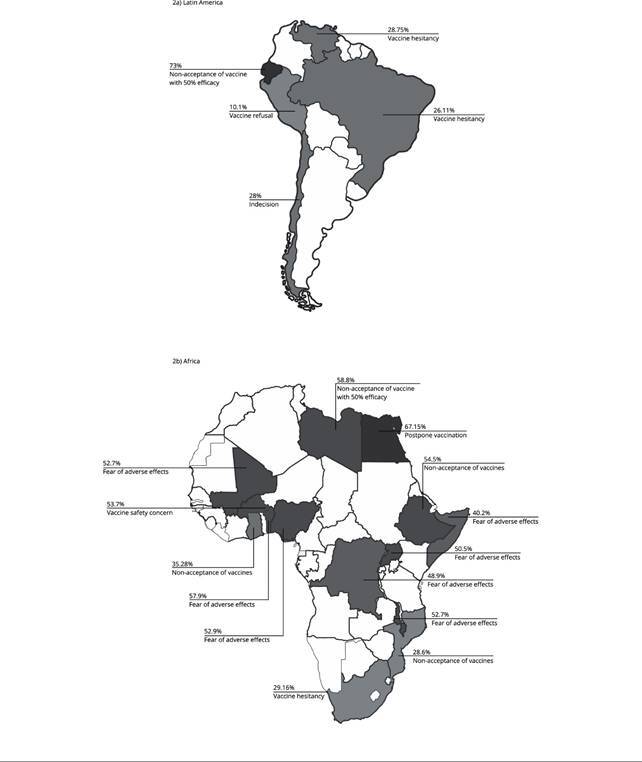
Highest percentages of COVID-19 vaccine hesitancy in studies in African and Latin American countries according to the nomenclatures used by the respective authors.

Source: prepared by the authors.

In Latin American countries, the highest vaccine hesitancy rate of 26.1% ^38^ and the lowest 8.4% ^40^ were reported in Brazil. In Ecuador, hesitancy ranged from 73% to 9%, depending on the vaccine efficacy ^14^. In Chile, 28% were hesitant ^39^ and 23% refused the vaccine ^39^. Peru had 10.1% refusal and 19.5% hesitancy ^41^. In Venezuela, vaccine hesitancy was 28.75% ^43^. Figure 2 shows a map with the highest percentages of COVID-19 vaccine hesitancy reported in selected studies.

The reasons for COVID-19 vaccine hesitancy were explored in 84 of total 94 studies (Box 2). The other studies did not specify the reasons in their results. The main reasons were: concern about possible adverse events (47.8%), safety issues of COVID-19 vaccines (31.9%), uncertainty about COVID-19 vaccine efficacy (34%), conspiracy theories (21.2%), lack of reliability in clinical trials/rapid vaccine development (15.9%), perception of the immune system as a better defense against COVID-19 than the vaccine (14.8%); religious beliefs (10.6%), lack of information about vaccines (10.6%), risk of contracting COVID-19 considered low (7.4%), being against vaccines in general (6.3%), vaccine cost (6.3%), and freedom of choice (2.1%).

**Box 2:** Main factors associated with COVID-19 vaccine hesitancy by continent and studies.

MAIN FACTORS	AFRICA	LATIN AMERICA
Adverse effects	Acheampong et al. ^11^; Adeniyi et al. ^97^; Adigwe ^26^; Aemro et al. ^98^; Agyekum et al. ^52^; Ahmed et al. ^37^; Alle & Oumer ^100^; Angelo et al. ^64^; Anjorin et al. ^24^; Anorue et al. ^104^; Bongomin et al. ^53^; Botwe et al. ^59^; Chinawa et al. ^107^; Dula et al. ^35^; Elhadi et al. ^34^; Fares et al. ^30^; Gbeasor-Komlanvi et al. ^111^; Iliyasu et al. ^54^ ^,^ ^113^; Kanyanda et al. ^25^; Khalis et al. ^115^ ^,^ ^116^; Mejri et al. ^119^; Mesele ^31^; Mose ^122^; Omar & Hani ^126^; Saied et al. ^130^; Shehata et al. ^132^; Solís Arce et al. ^133^; Tadele Admasu ^134^; Tagoe et al. ^135^; Taye et al. ^136^; Uzochukwu et al. ^137^; Zewude & Belachew ^141^	Alvarado‐Socarras et al. ^101^; Bagateli et al. ^61^; Cerda & García ^39^; Douine et al. ^109^; Jaramillo-Monge et al. ^14^; Stojanovic et al. ^40^; Vignier et al. ^138^; Vizcardo et al. ^41^
Uncertainty about vaccine safety and efficacy	Acheampong et al. ^11^; Agyekum et al. ^52^; Alhassan et al. ^32^; Alle & Oumer ^100^; Anorue et al. ^104^; Berihun et al. ^106^; Bongomin et al. ^53^; Botwe et al. ^59^; Carcelen et al. ^50^; Dinga et al. ^65^; Ditekemena et al. ^51^; Dula et al. ^35^; Gbeasor-Komlanvi et al. ^111^; Harapan et al. ^67^; Iliyasu et al. ^54^ ^,^ ^112^ ^,^ ^113^; Kanyanda et al. ^25^; Kanyike et al. ^60^; Kassaw & Shumye ^114^; Khalis et al. ^115^; Mejri et al. ^119^; Mesele ^31^; Mose & Yeshaneh ^123^; Orangi et al. ^127^; Saied et al. ^130^; Shiferie et al. ^58^; Tadele Admasu ^134^; Taye et al. ^136^; Uzochukwu et al. ^137^; Zewude & Belachew ^141^	Alvarado‐Socarras et al. ^101^; Bagateli et al. ^61^; Douine et al. ^109^; Jaramillo-Monge et al. ^14^; Macinko et al. ^62^; Rodriguez et al. ^15^; Ticona et al. ^38^; Vignier et al. ^138^
Conspiracy theories	Botwe et al. ^59^; Dinga et al. ^65^; Ditekemena et al. ^51^; Iliyasu et al. ^54^ ^,^ ^112^ ^,^ ^113^; Kanyike et al. ^60^; Oduwole et al. ^124^; Okafor et al. ^125^; Oyekale ^46^; Tagoe et al. ^135^; Uzochukwu et al. ^137^	Andrade ^42^ ^,^ ^43^ ^,^ ^103^; Chaves et al. ^47^; Gramacho & Turgeon ^48^; Jaramillo-Monge et al. ^14^; Paschoalotto et al. ^49^; Vizcardo et al. ^41^
Reliability of clinical trials/rapid development of vaccines	Adebisi et al. ^44^; Alle & Oumer ^100^; Berihun et al. ^106^; Botwe et al. ^59^; Fares et al. ^30^; Iliyasu et al. ^112^; Mesele ^31^; Shehata et al. ^132^; Shiferie et al. ^58^; Tagoe et al. ^135^	Douine et al. ^109^; Rezende et al. ^129^; Rodriguez et al. ^15^
Immune system capable of fighting COVID-19	Adebisi et al. ^44^; Agyekum et al. ^52^; Ahmed et al. ^37^; Bongomin et al. ^53^; Iliyasu et al. ^54^; Khiari et al. ^117^; Oyekale ^45^; Wiysonge et al. ^139^; Zewude & Belachew ^141^	Fares et al. ^30^; Moore et al. ^121^
Religious beliefs	Alhassan et al. ^32^; Alle & Oumer ^100^; Bongomin et al. ^53^; Botwe et al. ^59^; Orangi et al. ^127^; Tagoe et al. ^135^; Uzochukwu et al. ^137^; Zewude & Belachew ^141^	Andrade ^42^ ^,^ ^103^
Lack of information	Botwe et al. ^59^; Carcelen et al. ^50^; Fares et al. ^30^; Gbeasor-Komlanvi et al. ^111^; Mohammed et al. ^120^; Mose ^122^; Tadele Admasu ^134^	Cerda & García ^39^; Chaves et al. ^47^; Douine et al. ^109^
Low risk of COVID-19	Adigwe ^26^; Alle & Oumer ^100^; Chinawa et al. ^107^; Kanyanda et al. ^25^; Orangi et al. ^127^; Taye et al. ^136^; Wiysonge et al. ^139^	
Being against vaccines in general	Adeniyi et al. ^97^; Adigwe ^26^; Agyekum et al. ^52^; Anjorin et al. ^24^; Khiari et al. ^117^	Alvarado‐Socarras et al. ^101^
Vaccine cost	Adebisi et al. ^44^; Alle & Oumer ^100^; Anjorin et al. ^24^; Bongomin et al. ^53^; Dinga et al. ^65^; Harapan et al. ^67^	
Freedom of choice	Alle & Oumer ^100^; Mejri et al. ^119^	

Source: prepared by the authors.

### Particularities of vaccine hesitancy in the Global South

Although most studies are focused on quantitative data, some publications describe specificities in the Global South regarding vaccine hesitancy in social, cultural, political, and economic dimensions.

In the study conducted by Andrade ^42^ ^,^ ^43^, religious factors influenced vaccine hesitancy in Venezuela, where belief in conspiracy theories has increased with the country’s political instability. Also, non-religious participants were more willing to receive the COVID-19 vaccine than Catholic and Protestant participants, with Venezuelan Pentecostals as the most hesitant religious group regarding COVID-19 vaccines.

Regarding political factors, studies conducted in Brazil and Venezuela mentioned opposition to the vaccine of their respective presidents Jair Bolsonaro and Nicolás Maduro ^42^ ^,^ ^43^ ^,^ ^47^ ^,^ ^48^ ^,^ ^49^. Maduro questioned the safety of the AstraZeneca vaccine, even refusing to buy it, and because his government was not recognized by many nations ^42^ ^,^ ^43^. In Brazil, part of supporters of then President Jair Bolsonaro rejected the COVID-19 vaccine, based on Bolsonaro’s speech in relation to the vaccine as an individual choice and the criticism to the Sinovac-CoronaVac vaccine, produced by a Chinese pharmaceutical company ^47^ ^,^ ^48^ ^,^ ^49^. The negative perception of coping with COVID-19 and the political opposition to the Federal Government were associated with the intention to be vaccinated ^49^, in addition to the political context of delay in the acquisition and availability of COVID-19 vaccines and political disputes between federal and state governments ^47^ ^,^ ^49^.

The categories of race and ethnicity also influenced vaccine hesitancy - in Venezuela, marginalized ethnic minorities were more likely to present COVID-19 vaccine hesitancy ^42^ ^,^ ^43^. In South Africa, the black population showed lower vaccine hesitancy (26%) ^28^.

Regarding differences in vaccine hesitancy between urban and rural areas, findings from studies conducted in Zambia, South Africa, the Democratic Republic of the Congo, and Ghana showed that vaccine hesitancy was higher in urban areas with more access to the Internet and, consequently, to social media and misinformation about COVID-19 vaccines when compared to rural areas ^28^ ^,^ ^32^ ^,^ ^50^ ^,^ ^51^. In Nigeria, the population living in the south of the country was more likely to be vaccinated while the population in the north was more likely to refuse it ^24^. Then, strategies to reduce vaccine hesitancy must consider regional aspects of each African territory ^24^. In Latin America, the intention to be vaccinated in Peru and Brazil was lower in areas of greater social inequality ^38^ ^,^ ^41^.

Some epidemiological studies revealed that women were more likely to hesitate to accept COVID-19 vaccine in African countries ^24^ ^,^ ^25^ ^,^ ^28^ ^,^ ^32^ ^,^ ^36^ ^,^ ^37^ ^,^ ^52^ ^,^ ^53^ ^,^ ^54^ due to possible access to misinformation, such as the rumor that COVID-19 vaccine could make a person sterile ^37^.

In Africa, the history of resistance to vaccination and growing misinformation disseminated via social media by leaders and religious groups about vaccines in general, including COVID-19 vaccines, were addressed in some studies ^32^ ^,^ ^36^ ^,^ ^51^ ^,^ ^55^ ^,^ ^56^ ^,^ ^57^. The lack of clear information about the disease and vaccines were factors that influenced hesitancy in Ethiopia and the Democratic Republic of the Congo - with public distrust in participating in COVID-19 vaccine tests in the Democratic Republic of the Congo ^51^ ^,^ ^58^. Another factor that influenced vaccine hesitancy in African countries was the lower mortality from COVID-19 in these countries, due to the perception that the continent had a reduced risk of COVID-19, as in the case of Ghana and Uganda ^36^ ^,^ ^59^ ^,^ ^60^.

Two studies conducted in Brazil obtained a low percentage of COVID-19 vaccine hesitancy and a higher percentage of acceptance among respondents ^36^ ^,^ ^61^. According to these studies, the result is influenced by the high transmission and mortality rates of COVID-19 ^36^ ^,^ ^61^. However, another publication claims that hesitant participants did not understand or were not informed about the high risk of COVID-19 in Brazil ^62^.

The third aspects of this analysis emphasized the influence of the WHO SAGE group as a reference for designing epidemiological studies on vaccine hesitancy. The report produced by the group ^5^ ^,^ ^6^ and the publication by Larson et al. ^63^ presents tools to measure and monitor vaccine hesitancy such as the *Vaccine Hesitancy Scale* (VHS). Despite this effort, most epidemiological studies (88) did not use references, method designs, and instruments developed by the WHO SAGE.

Regarding the term “vaccine hesitancy”, 61 of the 94 studies mention it without referring to the WHO and 26 studies use the WHO definition in the introduction of the study, but do not discuss the results according to the WHO SAGE framework. One exception is the study by Anjorin et al. ^24^, conducted across the African continent, and whose corresponding author is affiliated with a research institution in South Africa. It provided the definition of vaccine hesitancy and used the 3C model as a reference to discuss the results. According to this study, the perceived risk of SARS-CoV-2 is significantly related to vaccine hesitancy; therefore, the authors concluded the findings agree with the model of confidence, complacency, and convenience proposed by the WHO SAGE ^24^.

Among the studies that used the scale or developed research instruments based on WHO SAGE publications (6), a study conducted in Ethiopia used a questionnaire to assess vaccine hesitancy of the participants according to the WHO definition ^64^. The WHO Matrix of Vaccine Hesitancy Determinants (contextual, individual/group determinants, and specific issues about vaccine/vaccination) was used in three studies - one in Brazil ^47^, one in Cameroon ^65^, and one in Egypt ^30^. Regarding the 5C questionnaire, a multicenter study conducted in Middle Eastern countries used an adapted version for the Arabic language and culture to investigate the psychological antecedents of COVID-19 vaccination ^66^. A multicenter study in Asia, Africa, and South America used the VHS to measure the belief in the benefits of vaccination and the perceived risk of new vaccines ^67^. All these studies had corresponding authors affiliated with institutions in the Global South.

## Discussion

COVID-19 vaccine hesitancy can be an obstacle to reducing the effects of the pandemic. The findings of this review show that concern about possible adverse events, uncertainty about vaccine efficacy and safety, and lack of confidence in clinical trials for the development of COVID-19 vaccines were similar to other studies ^16^ ^,^ ^17^ ^,^ ^68^ ^,^ ^69^. Considering the phenomenon of hesitancy is multidimensional, the main justifications for hesitancy involve factors that go beyond biomedical biases to include sociocultural aspects with dichotomies such as medical/scientific view vs. cultural/popular view and universality vs. singularity ^70^. This scenario became even more complex with the advent of COVID-19, with the resurgence of movements of disbelief in science, dissemination of fake news about vaccines, ideological polarization, and socioeconomic vulnerability ^9^.

The strong association between the political scenario and (non-)acceptance of vaccines is also reflected in COVID-19 vaccines. In this review, political instability, disbelief in the government and the health system, and the feeling of not having a voice or power in the face of structures such as the State itself, have a direct influence on the spread of conspiracy theories ^71^ ^,^ ^72^. On the other hand, it is important to critically analyze the scenario in which these conspiracy theories were created, as many of them have concrete roots in the recent local history of these territories.

Underdeveloped countries were repeatedly used for tests with human beings, which today resulted in vaccine refusal due to the fear of being laboratory subjects ^73^ ^,^ ^74^. The power relationship between the Global North and the Global South, expressed in a past of coloniality and violence still alive in the memory of colonized countries, is reflected in the rejection of practices that supposedly come from the North. Then vaccines are seen by different groups as population control strategies in underdeveloped countries, as “western malevolence”, or as a method to extinguish undesirable groups ^75^ ^,^ ^76^ ^,^ ^77^ ^,^ ^78^. Therefore, discussions that associate the low level of vaccine acceptance in Africa with the fact that Africa had lower COVID-19 mortality rates or more misinformation may lead to reductionisms ^9^.

On the other hand, associating low percentages of vaccine hesitancy with countries that had many COVID-19 cases and deaths may also disregard local contexts. This review, for example, found that many studies highlighted high acceptance of vaccine in Brazil, establishing this association. However, Brazil is internationally recognized for its National Immunization Program, which has built a culture of collective immunization ^79^ ^,^ ^80^. At the same time, like other Latin American countries - as seen in this review - the country had to handle political instability, mismanagement of the COVID-19 pandemic, denial speeches by the president of the republic, and well-grounded direct association between “being opposed to the government” and “intention to be vaccinated” ^47^ ^,^ ^48^ ^,^ ^49^.

Likewise, as demonstrated in this review, some countries in the Global South still face sanctions from Global North countries, due to the non-recognition of their governments - such as Venezuela ^42^ ^,^ ^43^. Then, the power relations are evident between the Global North and the Global South, requiring discussions on low vaccination coverage in these countries from a broad perspective, which does not reduce (non-)vaccination to vaccine hesitancy or lack of information ^9^ ^,^ ^81^.

Finally, in both Latin America and Africa, religious factors were also relevant in the population’s decision to be or not vaccinated. Religion is a driving factor for vaccine hesitancy in general in the Global South ^82^ ^,^ ^83^ ^,^ ^84^, and this trend was also seen for COVID-19 vaccines ^42^ ^,^ ^43^ ^,^ ^85^. Then, inserting religious leaders in vaccination campaigns can be beneficial for vaccine adherence ^86^ ^,^ ^87^ ^,^ ^88^.

Another important aspect in this review is the relationship between the studies and the publications of the WHO SAGE working group. Although WHO SAGE has establishes a definition for vaccine hesitancy, this term has been used in different ways in studies and this lack of conceptual clarity can lead to mistaken interpretations and generate confusion among researchers ^89^ ^,^ ^90^ ^,^ ^91^. Of note, the concept originally established for “vaccine hesitancy” has already been altered because of the resulting criticisms and reflections. In 2022, the BeSD working group proposed a new definition for vaccine hesitancy as a “*motivational state of being conflicted about, or opposed to, getting vaccinated; includes intentions and willingness*” ^10^ (p. VII).

Vaccine hesitancy can be used to explain concerns and questions about vaccination, the interval between the continuum between accepting and refusing all vaccines, used as a synonym for non-vaccination ^89^ ^,^ ^90^. On the other hand, because it has very comprehensive definitions and is used in studies with different population profiles, contexts, and explanatory factors, vaccine hesitancy can be considered a comprehensive category, and not an empirical concept ^92^.

Regarding the use of method designs and research instruments based on WHO SAGE publications, only 6 of all 94 studies in this review used these instruments. However, it should be noted that this review was conducted in January 2022, i.e., before the release of BeSD working group document. Even so, considering that other tools issued by the WHO were well established and validated, such as the Matrix of Determinants and the 5C scale, it is interesting that few studies have used them.

In the perspective of the Global Health, initiatives for the formulation of “global” policies and documents, based on the perspective and expertise of Global North countries and constantly defended by the WHO to be replicated in different contexts, have been criticized ^18^ ^,^ ^93^. Local specificities, for not allowing large-scale comparisons and implementation of policy and models and for requiring unique and adaptable responses, tend to be ignored ^93^. Top-down “one-size-fits-all” initiatives do not take into account living conditions and characteristics of the communities where they will be applied ^94^. Considering the above, the application of the vaccine hesitancy concept and instruments validated by the WHO may not be adequate to analyze issues of access to vaccines and cost in countries where vaccination is not universal.

### Study limitations

The limitations of this scoping review are related to the methodological stages of this type of study. Although a comprehensive search strategy was adopted, some relevant studies may not have been selected, such as technical studies and studies published in French, considering this language is spoken in some African countries. This review did not analyze how each study addressed hesitancy and acceptance in the questions of surveys and scripts of qualitative studies. In addition, the selected studies were not evaluated in terms of evidence quality, as the objective was to map studies on COVID-19 vaccine hesitancy in African and Latin American countries.

## Final considerations

The discussion about vaccine hesitancy and, more specifically, COVID-19 vaccine hesitancy, has been the subject of global discussion. The issues presented in this scoping review show that COVID-19 vaccine hesitancy in countries of the Global South is a complex phenomenon.

The use of instruments produced by the Global North can lead to a failure to understand the different social, cultural, and regional aspects involved in COVID-19 vaccine hesitancy, but these aspects are essential for further studies and implementation of health actions ^9^.

This scoping review showed that vaccine acceptance and hesitancy rates significantly ranged in different locations, which also indicates that particularities of these locations must be considered as different reasons for vaccine hesitancy. Also, most studies selected in this review are quantitative/epidemiological studies, which may also limit the understanding of vaccine hesitancy complexity in regional, local, and cultural aspects of African and Latin American countries. Then, qualitative studies in social sciences allow the analysis of thick description to understand the beliefs and attitudes that involve the phenomenon of COVID-19 vaccine hesitancy ^70^. In this sense, and based on the understanding of the Global South particularities, effective responses should be developed to address each particularity.
